# Perceived social support disparities among children affected by HIV/AIDS in Ghana: a cross-sectional survey

**DOI:** 10.1186/s12889-015-1856-5

**Published:** 2015-06-06

**Authors:** Paul Narh Doku, John Enoch Dotse, Kofi Akohene Mensah

**Affiliations:** Department of Psychology, University of Ghana, Legon, Ghana

**Keywords:** Perceived social support, Orphaned children, HIV/AIDS, Ghana

## Abstract

**Background:**

The study investigated whether perceived social support varied among children who have lost their parents to AIDS, those who have lost their parents to other causes, those who are living with HIV/AIDS-infected caregivers and children from intact families (comparison group).

**Method:**

This study employed cross-sectional, quantitative survey that involved 291 children aged 10–18 years in the Lower Manya Krobo District of Ghana and examined their social support disparities.

**Results:**

Multivariate linear regressions indicate that children living with HIV/AIDS-infected caregivers reported significantly lower levels of social support compared with AIDS-orphaned children, other-orphaned children and non-orphaned children independent of socio-demographic covariates. Children who have lost their parents to other causes and other-orphaned children reported similar levels of social support. In terms of sources of support, all children orphans and vulnerable children were more likely to draw support from friends and significant others rather than from the family.

**Conclusion:**

The findings indicate a need to develop interventions that can increase levels of social support for orphaned and vulnerable children within the context of HIV/AIDS in Ghana, particularly networks that include the family.

## Background

The lack of access to antiretroviral treatment and the protracted and latent nature of HIV/AIDS mean that increasing numbers of children will have to spend much of their childhood years with an infected parent or as an orphan [1, 2]. Parental illness or loss of parents permeates all aspects of a child’s life and often marks the beginning of a drastic change in their lives. Parental deaths and illnesses are childhood traumatic events that are associated with several physical, psychiatric and psychosocial health problems [3]. Prior work have highlighted that children orphaned by AIDS are at risks for a range of adjustment difficulties including emotional problems [1,4], behavioural difficulties [5], self-esteem [6], suicide ideation [7], anxiety [8, 9], conduct problems [1, 10], post-traumatic stress disorder [1, 4, 11], delinquency problems [12, 6, 1, 13].

HIV/AIDS also results in loss of social and family support with direct consequences for children [14,15]. Social support is found to be associated with psychiatric and mental health outcomes [16]. Availability or perception of social support is suggested to enhance the coping skills of children to handle stressing life events [17] and functions to reduce distress and psychiatric difficulties [18]. Social support could therefore, be a cost effective critical resource [19] that buffers the effects of mental illness among children [20]. The availability of support for children in communities affected by HIV/AIDS would vary with the prevalence and maturity of the epidemic [4]. The social support system is usually sustained by family relatives, neighbours and friends. In high prevalence and matured HIV/AIDS endemic countries like those in Southern Africa, HIV/AIDS multiple losses such as the death of parents, siblings, relatives and neighbours [21] may overwhelm the traditional support system provided by extended family members and other established supportive environments of community networks [22]. It is no surprise that some researchers suggested that the traditional support system is collapsing in such regions because of the HIV/AIDS crises [23–26].

However, in a low prevalence epidemic context like Ghana, there may still be available family and community support to mitigate the impact of the disease on children’s psychological wellbeing [27]. Ghana has an HIV/AIDS prevalence rate of 3 % [24]. Families in low prevalence regions could show considerable resilience in absorbing orphans [28, 29]. However, difficulties in expanding antiretroviral treatment and the high levels of stigma that are attached to the disease in low prevalence areas would result in isolation of infected family members [30] and unwillingness on the part of relatives to offer support when needed. Difficulty in securing a foster parent or caregiver for AIDS orphans were reported in low prevalence regions [5]. Other investigators also documented a decrease in care-giving for AIDS orphans and vulnerable children in both Kenya and Ghana [31]. Clearly, children affected by HIV/AIDS may lack social support compared to non-orphans [1]. Delva and colleagues also confirmed the sparse social network of friends and low social support from the family for AIDS orphans in Guinea [5]. Social support is located within local cultural and social contexts, and is often impacted by education, church activities, extended family and community members [32,21,33,34]. Children who receive adequate support from family, peers and others adapt well psychosocially while those who do not become depressed, lonely and withdrawn [35]. In the face of the evidence on the association between social support and psychiatric illnesses, there is urgent need to strengthen the social support system of orphans and vulnerable children within specific contexts to alleviate psychiatric and psychological distress with high cultural variability. Recently an intervention study in Rwanda found that community based youth mentorship enhanced community connectedness and social protection for children [19]. However, it is the argument of this paper that a better understanding of the levels and sources of social support, and differences in access to social support among orphaned and vulnerable children, could facilitate strategies to support these children and enhance their psychiatric wellbeing. Yet, no studies to our knowledge have assessed levels or sources of social support among AIDS-orphaned children as compared with other orphaned and non-orphaned children either in Ghana or elsewhere. This article aims to (a) assess levels of social support among children in a low HIV endemic context, Ghana, (b) assess sources of social support (e.g., support from family, friends, or significant others) among these children, (c) compare whether AIDS-orphaned children have different levels and sources of social support as compared with other-orphaned and non-orphaned children, and (d) to examine social support as contextual resilience factor in developing appropriate interventions for families affected by HIV/AIDS in Ghana [36]. The present paper utilizes Bronfenbrenner’s Social-Ecological Model as a guiding theoretical framework to achieve these aims. The model can be considered a meta-theory because it combines individual theories (biological and behavioural), interactional theories (attachment and family theories) and social theories (socioeconomic and social support theories) in explaining development and human behaviour. It offers a multidimensional perspective of influences on behaviour and helps to understand the multifaceted nature of risks and resilience factors.

## Method

The study was conducted in the Lower Manya Krobo District of Ghana. The district, found in the Eastern Region of the country is situated 88 km North-East of Accra (national capital). Currently, it is estimated that the district has 154,301 people, of which approximately 79,047 are females [37]. The main economic activities of the Krobos are farming and bead-making but due to shortage of lands, poor rainfall and subsequently low harvests, most of the people are now unemployed or at best underemployed. The majority of the Krobo people consider themselves as Christians (Teye 2005) but traditional beliefs and practices are widespread. The majority occupants of the district, the Krobos, have certain distinct cultural practices such as the traditional practice of “Yosedofiemi”-whereby a man must present certain items, including drinks and money, and he must also publicly perform a traditional dance following the death of either the mother-in-law or the father in-law, patrilineal inheritance system and “Dipo”-a highly celebrated rites of passage into adulthood for girls. Attempts by Christianity, education and modernity to end these practices were largely unsuccessful leading to rather altered and adverse changes. For example, the Krobos have now reduced the age when the girls go through the rite to as low as 5 years when the young girls are powerless. It is suggested that the present performance of dipo at such tender age is contributing to the increased rate of unprotected, premarital sex among the Krobos and its associated HIV/AIDS and other STI’s infections [24, 37]. Based on a study conducted in 2006, the total number of Orphaned and Vulnerable Children (OVC) was estimated at 6 500 [37].

The inclusion criterion for the present study is a child between 10 and 17 years. The OVC group includes a child who is 17 years or below and has either lost at least a parent or is living with HIV/AIDS-infected parents. For the “non-OVC group” participants must not have had HIV/AIDS related/other terminal illness or death in their families/households over the past one year. AIDS-orphan is defined as children who have lost at least one parent to AIDS. The latter term is used interchangeably as children orphaned by AIDS or AIDS-orphaned children. Institutionalised and foster children were excluded. Social support entails the provision of both material and non-material needs for children orphaned by AIDS and other causes [11]. In the present study it represents one’s score on the Multidimensional Scale Perceived Social Support (MSPSS).

### Participants and procedures

The population for the present study comprises of all OVCs (6500) within the Lower Manya Krobo District of Ghana. The present study was designed and conducted as community based cross-sectional survey design utilizing questionnaires. There was a pilot preceding the present study to validate (examine the appropriateness and comprehensibility of) the study instruments in the present research setting. Results from the pilot study demonstrated that the items in the questionnaire were easy to read, comprehend, psychometrically sound, non-distressing and relevant to the Ghanaian children. Participants were identified through multistage cluster sampling. First, the 54 communities within the district were stratified according to residential clusters, namely rural and urban clusters, based on existing geographical and population data [37]. This was intended to reflect the diversity of socio-demographic status of the participants. Secondly, communities were randomly selected from each cluster for participation in the study. Thirdly, a simple systematic sampling technique was used where starting from a known central point in each randomly selected community, every sixth household in respective directions (East, West, North, and South) was visited and screened for eligibility, and then household members identified the primary child caregiver in the home.

However, there were problems in obtaining enough data from participants using the multi-stage cluster sampling technique. In the rural communities, the settlements were unplanned and distances between households were found to be lengthy. Another recruitment method, total sampling, was therefore employed to ensure higher participation. Thus the multi-stage sampling technique was changed mid-way through the fieldwork data collection due to practical issues encountered in favour of a total sampling technique whereby every household in selected communities were approached and asked to participate in the study. This sampling procedure is justified for reasons of feasibility, cost-effectiveness, and accessibility. Then within each community, a limit of 30 was placed on the number of households recruited. This was done to minimise the effects of over-representation from particular communities. Data gathered from both recruitment strategies were combined in the final data for analysis since preliminary analysis found them similar on key variables. Each participating child completed a survey questionnaire that followed the steps described by Thomas [38]. The entire assessment inventory took about 30 to 45 minutes to complete. Written informed consent for participation in the study was obtained from the participants. However for children below 15 years, a parent or guardian provides the written informed consent. The study protocol was approved by the Institutional Research Ethics Review Boards of the University of Glasgow and the Ghana Health Service prior to recruitment of children.

If at least one child in the house was an AIDS orphaned child, then the child was classified as an AIDS-orphaned child and recruited as such to handle issues of multiple eligible OVC. Verbal Autopsy (VA) was used to identify whether children lost one or both parents from AIDS [39]. The present study used the verbal autopsy method to assess the cause of parental death because of the difficulty of obtaining accurate death certificates and because caregivers were often unaware of or did not wish to disclose the parental cause of death. The VA method uses reports on eight signs and symptoms of HIV to verify cause of death. The study used a conservative endorsement of at least 6 HIV/AIDS-defining symptoms compared to the 3 that were often used in earlier investigations [1]. The original study had a sensitivity of 66 % and specificity of 76 % of predicting death due to AIDS; sensitivity and specificity did not vary significantly according to gender, time of death, and whether the respondent was a primary caregiver, family member, or other relation to the deceased [40, 41]. Verbal autopsy validation in Ghana also compared assigned causes of death from verbal autopsy to medically certified causes of death [40] and was found to correctly identify 78 % of deaths. Verbal autopsy has since been used in several studies with high predictive powers [42–44]. The World Health Organisation and Health Metrics Network have approved it for use in developing countries and asserted that it is an essential means of ascertaining AIDS deaths in the absence of HIV/AIDS sero-status record of deceased persons [45]. Lopman and colleagues state clearly the value of verbal autopsy: it is the only option to identify cause of death in widespread HIV/AIDS epidemic settings [39, 41].

### Measures

Social support was assessed using the Multidimensional Scale Perceived Social Support (MSPSS) [46]. This scale consists of 12 items measuring perceived social support from the family, friends and significant others. The scale is rated on a 5-point likert type ranging from strongly agree =5 to strongly disagree =1 with negated (no) items reversely scored. The scale yields 4 perceived social support scores: Family, Friends, Significant Others and Total. The MSPSS internal reliability were .91, .87 and .85 Cronbach’s coefficient alpha for support from significant others, family and friends respectively [47]. The present study found similarly high Cronbach’s coefficient alpha of .80, .86, .91 and .88 for support from family, friends, significant others and total (full scale) respectively.

A number of socio-demographic factors such as age, gender, family size, number of other minors living at home, number of changes in residence and age at which children were orphaned (where applicable) were measured using items from the Demographic and Health Survey Questionnaire [48]. There were also items regarding children’s educational level as well as their present education status (presently at school or not).

### Statistical analyses

Data were analyzed using SPSS version 18. The analysis involves three key factors: perceived social support, socio-demographic factors and orphanhood groups. The analyses followed four key steps. First, the differences between the orphanhood groups on socio-demographic factors were established using chi-square tests or analyses of variance (ANOVAs). Second, the relationships between the various socio-demographic factors and the perceived social support were examined. These were assessed using independent t-tests and chi square (for categorical variables such as gender, religion and being presently in school) and Pearson bivariate correlations, and ANOVA (for continuous socio-demographic such as age, household size and number of children at home). Third, linear regressions were performed to develop models that investigated the association between orphanhood types and perceived social support without controlling for any socio-demographic factors (unadjusted models). These were done independently for children orphaned by AIDS, other orphans and living with HIV/AIDS-infected parents, compared to non-OVC. Since perceived social support was predicted by orphanhood groups, the fourth step we conducted adjusted multivariate linear analyses (adjusted models). These second models examined the association between orphanhood groups and perceived social support when controlling for all relevant socio-demographic co-factors. These models established whether perceived social support among the orphanhood groups persists independently of socio-demographic co-factors. Inclusion of socio-demographic co-factors into the models was guided by their significant association (p < .05) with the perceived social support or significant differences between the orphanhood groups [1]. Based on using General Linear Models involving 8 predictors and working at .05 level of significance, the G Power estimated that a minimum sample size of 59 children in each group of the 4 groups (237 in total) was sufficient to obtain power of 95 % to detect a small effect (.10 effect size).

## Results

### Socio-demographic characteristics of participants

The 291 participants involved in the present study had a mean age of 13.03 years (SD = 2.87), with age range 10–18, there were 51 % female, and ethnic origin was 63 % Krobos. There was an average of four people living in the household. The majority of the children (81.8 %) were currently attending school. About 75 % had attained primary or junior secondary level education and 12.7 % vocational or technical education. Concerning parental/caregiver educational level, approximately 58 % of them had no more than senior secondary level education. Overall, 62 % of all children had changed residence between two or more times (55 % of non-orphans, 53 % of AIDS-orphans, 85 % of other-orphans and 56 % of HIV/AIDS-infected parents). The majority of parents and caregivers (62 %) worked mainly in farming, driving, trading or as artisans (carpentry, masonry, bead making). Eleven percent of parents worked in the formal sector (employment which offer regular wages and hours, which carry with them employment rights, and on which income tax is paid) whilst approximately 13 % of them were unemployed. The proportion of households with unemployed parents was higher among children living with HIV/AIDS-infected parents (38 %) than AIDS-orphans (9.5 %), other-orphans (9 %) and non-orphaned children (7 %). In the sampled about 56 % of the children (69 % of non-orphans, 49 % of AIDS-orphans, 45 % of other-orphans and 56 % of those living with HIV/AIDS-infected parents) indicated that they were Christians, 11 % Islam, 20.3 % Traditional/African beliefs and 12.7 % belonging to other faith. Descriptive statistics of the participants are summarized in Table 1.

**Table 1:** Socio-demographic characteristics of the study sample

	Non-orphaned and vulnerable children (n = 100)				P value (*t*-test/chi-square)
		AIDS-orphaned vulnerable children (n = 74)	Other-orphans (n = 67)	Children with HIV/AIDS infected parent/caregiver (n = 50)	
Age	11.53 (2.683)	13.78 (2.624)	13.09 (2.673)	14.84 (2.324)	F = 21.131^c^
Gender: Girls	52	50	50.7	48	
Boys	48	50	49.3	52	n. s.
Ethnicity: Dangme/Krobo	63.0 %	59.5 %	73.1 %	56.0 %	X = 40.051^c^
Presently schooling	91.2 %	74.1 %	84.0 %	72.4 %	X = 11.987^c^
Household size	4.98 (0.995)	3.73 (0.969)	4.27 (1.226)	3.96 (1.068)	F = 22.604^c^
No. of changes in residence	1.35 (1.336)	2.76 (1.524)	3.09 (1.685)	1.72 (1.471)	F = 23.844^c^
No. of siblings	1.21 (0.946)	1.95 (0.935)	2.22 (1.277)	2.44 (1.198)	F = 19.807^c^
Location where child lives: urban	50.0 %	60.8 %	59.7 %	58.0 %	n. s.
Age child first bereaved		6.27 (4.339)	8.81 (3.456)		
Parental educational level: > Junior Secondary					
Parental unemployment	7.0 %	9.5 %	9.0 %	38.0 %	X = 39.695^c^
Parental Loss: Mother	-	33.8 %	34.3 %	-	n. s.
Father	-	37.8 %	41.8 %	-	
Both	-	28.4 %	23.9 %	-	
Religion: Christianity	69.0 %	48.7 %	44.8 %	56.0 %	X = 36.271^c^

^a^Denotes significance at the 0.05 level, ^b^Denotes significance at the .01 level, ^c^Denotes significance at the .001 level

ANOVA shows significant differences [F (287, 3) = 21.131; p < .001] in the age between the four groups. Non-orphaned children were younger than AIDS-orphaned (t = 2.254, p < .001), other-orphans (t = 1.560, p < .001) and children living with HIV/AIDS-infected parents (t = 3.310, p < .001). Similarly, children living with HIV/AIDS-infected parents were older than AIDS-orphaned children (t = 1.056, p < .05) and other-orphans (t = 1.750, p < .001), with no other differences between groups. Other-orphaned children (Mean = 8.81, SD = 3.456) were older than AIDS-orphaned (Mean = 6.27, SD = 4.339) at the time of first bereavement [t (df = 134) = 2.751; p < .01].

The data also indicated different levels of household size. Non-orphaned children are living in households larger than AIDS-orphans (t = 1.250, p < .001], other-orphans (t = 0.711, p < .001) and children living with HIV/AIDS-infected parents (t = 1.020, p < .001). Other-orphaned children live in household size larger than AIDS-orphaned children (t = 0.539, p < .01) with no other differences between groups. There were also significant differences between the orphanhood groups on movement/changes in residence [F (3, 287) = 23.844, p < .001]. Other-orphaned children have moved between homes more than children living with HIV/AIDS-infected parents (t = 1.370, p <. 001) and non-orphaned children (t = 1.740). Similarly, AIDS-orphans have also moved between homes more than children living with HIV/AIDS-infected parents (t = 1.037) and non-orphaned children (t =1.407), both at p < .001 with no other differences between groups. Socio-demographic differences are summarized in Table 1.

### Differences between OVC groups on perceived social support (PSS)

Overall levels of total perceived social support were high among the sample, and showed significant group differences [F (3, 287) = 53.688, p < .001]. A follow up post hoc multiple comparison indicate that the level of perceived social support was highest among the non-OVC, lowest in the children living with HIV/AIDS-infected parents, and both AIDS orphaned children and those children orphaned by other causes were in the middle. Concerning the subscales of the perceived social support scale, there were no statistical significant differences in support from friends [F (3, 287) = 1.406, p = n. s.] and support from others [F (3, 287) = 1.964, p = n. s.] among the four orphanhood groups. However, at significance level of p = .05, it was found that AIDS orphaned children have lower perceived social support from significant others than non-OVC (0.626, p = .049).

Examining the family subscale of the PSS scale, significant group differences were observed in a one-way ANOVA analysis. Post hoc comparison revealed that non-OVC comparison children reported higher levels of support from family than children orphaned by AIDS (t = 4.638, p < .001), who also reported higher social support from friends than both children orphaned by other causes (t = 0.972, p < .01) and those living with HIV/AIDS-infected parents (t = 0.922, p < .01). The levels and sources of social support are summarised in Table 2

**Table 2:** Levels and sources of social support and differences between the four groups

Source	Comparison group of children (n = 100)	Orphaned and vulnerable children			F
		AIDS-orphaned children (n = 74)	Other-orphans (n = 67)	Children with HIV/AIDS-infected parents (n = 50)	
PSS Scale (Total Support)	38.18 (4.71)	33.00 (3.24)	32.15 (3.45)	31.26 (3.29)	53.685^c^
FAMILY	12.76 (2.31)	8.12 (1.78)	7.15 (1.73)	7.20 (1.67)	160.226^c^
FRIENDS	12.78 (2.47)	12.86 (2.18)	12.46 (2.52)	12.06 (2.39)	1.406
OTHER	12.64 (2.18)	12.01 (1.81)	12.54 (1.93)	12.00 (2.37)	1.964

^c^Denotes significance at the .001 level

.

### Association between socio-demographic factors and social support

Some socio-demographic factors were found to correlate with social support. Older age was found to be associated with lower perceived social support (r = −.228, p < .001). There was no difference observed according to gender (t = 0.605, p = n. s.). Lower perceived social support was also associated with living in smaller households(r = .206, p < .001), having more siblings (r = −.228, p < .001) and not currently attending school (t = 2.100, p < .05).

### Levels of social support after adjusting for socio-demographic factors

Multivariate linear regressions were conducted with the overall social support score as dependent variables. Because of the significant patterns of difference that children who have lost their parents to AIDS, those who have lost their parents to other causes, those who are living with HIV/AIDS-infected caregivers and children from intact families showed in levels and sources of social support compared with the other groups in bivariate analyses, multivariate models focused on comparing each of these OVC groups with non orphaned children (comparison group). Unadjusted and adjusted models for these analyses are presented in Table 3.

**Table 3:** Multivariate linear regression associations between orphanhood by AIDS, orphanhood by other causes, living with HIV/AIDS-infected parents versus non-orphaned children on social support controlling for socio-demographic co-factors

Source	Orphaned by AIDS Vs non-orphans		Orphaned by other causes Vs non-orphans		Living with HIV/AIDS infected parent Vs non-orphans	
	Unadjusted Model (β)	Adjusted Model (β)	Unadjusted Model (β)	Adjusted Model (β)	Unadjusted Model (β)	Adjusted Model (β)
	157^c^	-.138^b^	-.244^c^	-.218^c^	-.288^c^	-.208^c^
Gender		-.052		-.054		-.046
Age		-.135^a^		-.156^a^		-.090
Presently schooling		.071^a^		.082^a^		.020
Household size		.190^c^		.199^c^		.174^c^
Changes in residence		-.037		-.018		-.095
Number of siblings		-.183^c^		-.183		-.145^c^

^a^Denotes significance at the 0.05 level, ^b^Denotes significance at the .01 level, ^c^Denotes significance at the .001 level

In the unadjusted model being orphaned by AIDS was significantly associated with lower levels of social support (β = −0.157, p < 0.001). In the final adjusted model that controlled for relevant socio-demographic variables, AIDS-orphaned children still reported significantly less social support compared with non-orphaned children (β = −0.138, p < 0.001). The final adjusted model also identified age (β = −0.135, p *<* 0.05), presently in school (β = 0.071, p *<* 0.05), household size (β = 0.190, p *<* 0.001) and number of siblings (β = −0.183, p *<* 0.001) as an important socio-demographic covariate for levels of social support among AIDS-orphaned children.

In the unadjusted model being orphaned by causes other than AIDS was significantly associated with lower levels of social support (β = −0.244, p < 0.001) compared with non-orphaned children and this association was still significant in the adjusted model that controlled for relevant socio-demographic variables (β = −0.138, p <0.001). The adjusted model identified age (β = −0.156, p *<* 0.05), presently in school (β = 0.082, p *<* 0.05), household size (β = 0.199, p *<* 0.001) and number of siblings (β = −0.153, p *<* 0.001) as an important socio-demographic covariate for levels of social support among other-orphaned children.

Finally, living with an HIV/AIDS-infected parent was significantly associated with lower levels of social support in the unadjusted model (β = −0.288, p < 0.001) compared with non-orphaned children. In the final adjusted model that controlled for relevant socio-demographic variables, children who were living with an HIV/AIDS-infected parent still reported significantly less social support compared with non-orphaned children (β = −0.208, p < 0.001). The final adjusted model also identified household size (β = 0.174, p *<* 0.001) and number of siblings (β = −0.145, p *<* 0.001) as important socio-demographic covariate for levels of social support among children living with an HIV/AIDS-infected parent.

## Discussions

The analyses found significant differences between the orphanhood groups on perceived social support from the family but not from friends and significant others. Concerning support from the family, children living with HIV/AIDS-infected parents and other orphans reported the least perceived social support consistent with [1,31]. Comparison children reported higher perceived social support from the family than children orphaned by AIDS. That is, whilst the family may be the main source of emotional support for comparison children that may not be the case for children affected by HIV/AIDS. Our findings suggest a weakening of the traditional support system provided by families where households are affected by HIV/AIDS in the Manya Krobo District. The fact that children affected by HIV/AIDS reported large household sizes yet lower social support from the family suggests that the family members are there but are simply not supportive rather than a notion of a diminished family in the presence of HIV/AIDS.

It is particularly interesting that children living with HIV/AIDS-infected parents reported lower perceived social support than children orphaned by AIDS. Several factors could account for this situation. First, it might be that parents-infected with HIV/AIDS are preoccupied with their own situation (HIV/AIDS infection and its attendant stress) to the extent that it compromises their ability to provide quality child care, yet other support systems have not come into play because the parents are still alive and in a care-giving role. Okawa and colleagues suggested that when a parent is infected with HIV/AIDS, it becomes difficult for him/her to provide adequate care for children [32]. Similarly, as infected parents progress with the HIV/AIDS in severity, they may become too ill to be available for support. The present study did not capture ART among infected parents but it will be interesting to have future research comparing those not receiving ART with those receiving ART which would control for HIV in the family but compare well versus ill parents.

Second, when a parent is infected with HIV/AIDS, other family members may shift the available support to the sick parent to the detriment of the child, thus further limiting the support available to the child. Third, it is also possible that most children when orphaned by AIDS are well cared for by the surviving parent, extended family or others, whereas in the case of children living with HIV/AIDS-infected parents, parental sickness more severely reduces and limits the availability and quality of support for children [49]. Fourth, living with HIV/AIDS-infected parents, places care responsibilities on children and this may affect their social support network. Thus, children living with HIV/AIDS-infected parents may assume care-giving responsibilities for their parents and young siblings, restricting their opportunity to find support from elsewhere (e.g. school). This might not be the case with AIDS orphaned children [4]. Fifth, in Ghana, the limited existing interventions from the government and non-government organisations for children affected by HIV/AIDS exclude children living with HIV/AIDS-infected parents.

Just as in many other countries affected by the HIV/AIDS pandemic, AIDS orphanhood is often the primary criterion used by organisations for eligibility for HIV/AIDS interventions for children. This could account for the finding that children orphaned by AIDS reported higher perceived social support than those living with HIV/AIDS-infected parents. Finally, as reported earlier, children living with HIV/AIDS-infected parents experience more stigma, discrimination and social exclusion than other children even those whose parents have died from AIDS. This could limit their available social network and affect their ability to seek and receive support from their family, friends and the community at large. Supporting this speculation, a recent study in China showed that high levels of AIDS related stigma were related to lower perceived social support [22]. Other researchers also suggested that stigma associated with poverty and HIV/AIDS often impacts on one’s available social support network [34].

The findings of the present study indicate that all children, regardless of their vulnerability status, draw primarily from friends and significant others, rather than the family, for support. These results have important implications for interventions aimed at strengthening social support available to these children. Such findings indicate that efforts to bolster orphaned and vulnerable children’s social support networks should focus on significant others and friends rather than family. Certain socio-demographic factors also showed significant associations with social support that may be important in developing developmentally and contextually appropriate interventions that would enhance social support among children affected by HIV/AIDS.

First, currently attending school is a significant predictor of higher perceived social support from friends and significant others. This indicates that keeping children in school may offer them an opportunity to generate a larger social network that might enhance their social support and emotional wellbeing. This is consistent with observation in Uganda that schools provide OVC with comfort and relief from distress and grief as they spend quality time with school mates and responsible teachers [21]. Okawa and colleagues also noted in urban Kenya that, when parents have the funds to keep children affected by AIDS in school, the parents are not only paying for formal education but for the children’s emotional care too [32]. Clearly, educational institutions and opportunities are important avenues to promote the social support network of children as they offer normality and stability for OVC [20]. In Rwanda, it was demonstrated how youth mentorship conducted in schools enhanced community connectedness and social protection [19]. The school offers a place where teachers can be mobilised and empowered to identify children affected by HIV/AIDS and offer or recommend specific psychological interventions in addition to more traditional social support.

Second, larger household size was associated with higher perceived social support. Thus living in families with larger numbers of adults seems to enhance children’s perception of social support. This suggests that children affected by HIV/AIDS would receive better emotional care if they are placed in households with more adult members or relatives. Perhaps adult relatives would contribute their support, however little, to provide care and comfort. This suggests that adult family members and relatives are the closest people who could understand the psychological difficulties children affected by HIV/AIDS experience and may provide opportunities for them to connect with other relevant support networks.

Third, in the present study, living with many siblings in the same households was related to lower perceived social support. In an earlier study it was found that living with siblings enhances perceived social support and advocated for siblings affected by AIDS to be placed together [32]. Yet other investigators suggested the contrary that children orphaned by AIDS are worse off as regards their mental health when they are separated from their siblings [22]. The present findings, however, suggest that whilst siblings may provide vital means of social network and connection, it may not be beneficial if several siblings are placed together. One reason could be that living with many siblings places care responsibilities on each other and this may affect their social support network. It is therefore suggested that large siblings should not be placed together during HIV/AIDS parental illness or after parental death.

Finally, increased age was significantly associated with lower perceived social support. A speculative reason for this association may be that older children affected by HIV/AIDS assume the responsibilities of providing care and support for young siblings in ways that may affect the quality of support that they themselves receive [4, 27] and are less likely to be in school. Thus the significant differences and associations of perceived social support across age, orphanhood types, household size, number of sibling and children’s present educational status call for developmentally appropriate and context specific interventions for effective enhancement of social support.

The current study presents several potential limitations. Firstly, the relationship between psychosocial adjustment and perceived social support was based on retrospective, self-reported, cross-sectional data, which limits the causal interpretation of the findings. Longitudinal data research is needed to understand their relationship better. Secondly, this is the first time that the perceived social support scale (MPSS) is used in Ghana. It was not previously validated for sound psychometric properties and had relatively low reliability estimates in the current study. Furthermore, the scale could not distinguish between availability (quantity) and quality of social support. Future studies are needed to develop reliable measures of social support that are culturally and developmentally appropriate for children affected with HIV/AIDS, and can assess both quantity and quality of social support available. Thirdly, the present study was quantitative and utilised structured questionnaires. As typical of any structured measurements, the study is limited to only outcomes that were predetermined and included in the design of the study. The design did not allow for other outcomes to be added in the course of data collection. Fourthly, the unavailability of blood serology tests of parental HIV/AIDS status compelled the study to rely on self-reported cases of HIV/AIDS-infections. This method of parental HIV/AIDS status assessment is spurious because just like many other African societies, in Ghana people are not open about HIV/AIDS and HIV/AIDS testing is very low in Ghana; hence there was the possibility that some of the families classified as comparison group were in fact HIV/AIDS sufferers. Additionally, the HIV/AIDS status of the children themselves was not taken into consideration although there is evidence that this might impact and compromise children’s wellbeing [1]. The study also relied on verbal autopsy, ie information from close relatives to identify causes of adult death because there was essentially non-existence of reliable medical information (e.g. death certificate) on adult mortality. The verbal autopsy method of death identification has been validated in several studies in African contexts, including Ghana, where the present study was conducted [28,39]. Another limitation relates to the characteristics and nature of the sample used in the study that may not guarantee the generalisation of the findings. The sample size, although larger than those used in most earlier studies, is modest and came from just one out of the 130 districts in Ghana. Caution needs to be taken in attempting to generalise the findings of the study to the larger population of Ghanaian children. Finally, other important factors such as stigma and poverty that could impact one’s access to social support were not captured to be included in the statistical analysis. The findings of the present study should therefore be cautiously understood in the light of these caveats.

## Conclusions

Despite these limitations, the present study shows several strengths and makes significant contributions to the evidence base about children affected by HIV/AIDS in relation to their levels of social support. In sum, findings certainly make an important contribution to the literature. This is the first study to our knowledge to highlight levels of social support among orphaned and vulnerable children within the context of HIV/AIDS as compared with non-orphaned children in Ghana. It is also the first study to our knowledge to identify whether sources of social support differ between AIDS-orphaned children, other-orphaned children, children living with HIV/AIDS-infected caregivers and non-orphaned group. This study contributes to our knowledge of how to support OVC and also identified the vast scope for further work in this area, as discussed above. In order to effectively care for these children, families in HIV endemic communities must have social resources [50]. Policies and programs may be able to strengthen the resilience and improve the wellbeing of families caring for these children by addressing disparities in social support among children affected by HIV/AIDS, and by building on existing sources of social support for these children, including family, significant others, and the broader community. These findings strongly indicate a need for more generalized support for all children living in HIV prevalence areas, suggesting that intervention approaches should not single out specific population subgroups or households in a way that may further deepen their social seclusion and stigma. Indeed, as our analyses highlight, there is an urgent need to support all orphaned and vulnerable children and their households and to ensure that they are included and prioritized within policy and program responses.
